# Horseback riding pathways and harbors at the beginning of the colonial era in Mexico

**DOI:** 10.1038/s41598-020-67523-3

**Published:** 2020-06-26

**Authors:** Igor Lugo, Martha G. Alatriste-Contreras

**Affiliations:** 1 National Autonomous University of Mexico, Centro Regional de Investigaciones Multidisciplinarias, Estudios Regionales, 62210 Cuernavaca, Mexico; 2 National Autonomous University of Mexico, Facultad de Economia, Metodos Cuantitativos, 04510 Mexico City, Mexico

**Keywords:** Socioeconomic scenarios, Computer science

## Abstract

The introduction of horses in the New World changed the way of traveling on complex terrains. This change reconfigured the land transport network connecting harbors in the region. However, data of horseback riding pathways among harbors is scarce. We analyzed the case of Mexico at the beginning of the colonial period to recreate routes that connected ancient harbors and to identify the network characteristics of a large-scale system of routes. We used the complex systems approach as a framework in which we applied the least cost path analysis to reconstruct a network of horseback paths, and we computed the node betweenness centrality to identify the most probable locations that controlled de flow of travels. Findings suggest that horses modified the transportation system by expanding the connections and increasing the speed of traveling across the New Spain territory. The node betweenness centrality suggests that some locations organized the flow of traveling based on a few harbors located at the central region. Therefore, the horse allowed the Spaniards to reshape the spatial organization in the colonial era in Mexico.

## Introduction

Spanish introduced horses in the New World changing the way of traveling in the territory. In particular, from human walks to horseback riding, the connections between settlements and unexplored regions increased, and routes improved their speed of traveling. This change in the transportation technology accelerates the rate of progress because humans increase their mobility^1^. However, it is not clear how this fortuitous innovation in mobility reorganized the land transport network and connected trading ports in the New Spain. Following the conquest of the Aztecs, Early Colonial (1521–1620 CE) period,^2^ the New Spain searched for potential port sites because it had to guaranty the political and economical control of the region. The Spanish colonizers found ancient harbors that were used by different tribes long before the Aztecs These natural harbors were located across all the territory and were declared as part of the colony. However, historical data of how horseback pathways replaced the Aztec transportation system based on human carriers and connected ports in the New Spain are missing due to the lack of historical sources and experimental data.

An Initial attempt that focused on identifying the importance of horses in the New World is the work of Cunninghame and Moorman^3^. They suggested that the horses were the key to conquer the Aztecs and developed the New Spain due to horses’ physical performances, such as crossing almost any form of terrain and traveling short and long distance paths faster than walking speed. In addition to horses, we cannot forget the importance of donkeys and mules as cargo animals in the New Spain because they complement the horse’s work. For example, they transported silver—a convoy leading by a rider—to Caribbean ports for shipment to Spain^4,5^. For simplicity purposes, we analyze the case of horses even though these animals share physical advantages to move goods and humans in rugged areas and mountain terrains. These advantages were explored by Wickler et al.^6^ They suggested that the preferred speed of horses caring an additional mass—for example a rider—was the minimum when horses are trotting. Then, horses regulate the muscle stress producing longer endurance. This result is related to the work of von Lewinski et al.^7^ who suggest that horses do not get anxious due to riders’ stress behavior, but when the horses view the human facial expressions related to emotions, they understand them^8, 9^. Therefore, since the human domesticated horses—during the Bronze Age, 4,100 to 3,000 years ago^10^—this animal has created an empathy with humans and showed communication across species and terrain adaptability advantages.

With respect to ancient harbors and ports in Mexico, there are a limited data and historical documents to describe their location and their functions in particular contexts. For example, their locations are fundamentally related to ancient civilizations, i.e., there were natural harbors across all the territory long before the Spanish conquered the Aztecs. These locations are the key to understanding the trading mechanisms and the emergence of that we know as urban places or cities. Lugo et al.,^11^ found that ports and cities are related to each other due to their spatial proximity. In particular, the number of people in a city is related to its closest ports. Therefore, in the context of the colonial era, it is fundamental to describe the land connectivity of harbors since they had developed into port infrastructures based on the exploitation of metals, in particular the silver^12^.

In terms of the method, current studies have pointed out the necessity of using an interdisciplinary approach to understanding ancient transport networks. In particular, based on the archaeology approach, Verhagen et al.^13^ show the current knowledge in computer-based modeling of pathways and networks. They suggest that such models need to consider different perspectives and improve the sensitivity analysis and their validation. These suggestions were considered by Lugo and Alatriste-Contreras^14^ using the complex systems approach. They generated spatial networks based on the least cost path (LCP) method and compared them with theoretical and empirical networks. They found that spatial networks based on land transport modes show similar behaviors related to skewed statistical distributions of path lengths.

Therefore, the purpose of this study is to describe the full spatial network of horseback riding that connects harbors in the beginning of the colonial period. In particular, we reconstructed traveling routes based on the LCP approximation and applied the node betweenness centrality (NBC) measure to identify route sections that organized the flow of travels between harbors. We used an interdisciplinary analysis based on the complex systems approach to reconstruct and study spatial networks in ancient times. Especially, we used the concept of universality to describe a similar large-scale behavior in the land transport network even though it differs in the transport technology, for example humans, domestic animals, or wheeled vehicles. We would like to know what was the effect of horses in the land transport network of traveling routes at the beginning of the colonial period? And how horseback riding connected harbors based on pathways in the territory? To answer these questions, we used the geographic information system (GIS) technology, the field of animal physiology, the archaeology approach, and the complex network analysis. The GIS technology provided the geospatial approach to collect, organize, and visualize data; the animal physiology provided the experimental data to describe the horse behaviors as mathematical formulations; the archaeology showed the application of the LCP methods and contextualized pathways in the territory at specific time and scale; and the complex networks analysis identified the statistical distributions of data related to different spatial networks. In particular, we proposed a method that generates different spatial models based on the work of Self et al.^15^, Eaton et al.^16^, and Tobler^17^. We define these formulations as the training data set, and we compared them with a control data set based on the current road network of the region. We used the training and control data sets to apply a sensitivity analysis and validation of our spatial models. Both procedures used the concept of universality to identify similar or different statistical behaviors among data sets. The sensitivity analysis compares our proposed formulations to each other, and the validations compare these formulations with the current road network to test the accuracy between two different land transport networks located in the same region and terrain regardless of the gap between time periods. Next, we computed the NBC per formulation to understanding the contribution of route sections and harbors in the development of regional trade, and we showed their best fit of statistical distributions—cumulative distribution functions (CDF)—based on the Kolmogorov–Smirnov (KS) test for goodness of fit.

We believe that the introduction of horses in the New World expanded the number of routes to connect settlements strengthening the political and military control of Spain in the territory. This type of behavior was observed in the Mongol empire—the largest land empire in the history—in which the military advantage over sedentary armies was the use of riding horses^18^. Compared with human walking, horses improve the movement in traveling across complex landscapes and large distances. Then, horses decrease the energy expenditure and increase the movement speed of humans. We hypothesize that horses connected harbors based on a set of shortest routes that passed through the center of the region due to the importance of Tenochtitlan and surroundings. Therefore, the present work illustrates the structural change in transport routes when a new transportation technology is adopted. This change reorganized the exchange of goods, people, and information that affect the trajectory of the transport system producing the emergence of novel transport systems as the rail and road networks in years to come.

The scientific novelty of this study is related to recreate horseback paths at the beginning of the Colonial era based on limited physical and historical evidence. The importance of these paths is to understanding the transport network in which the flow of trade was going through the evolving harbors. The harbors supported the emergent social and economic organizations and subtracted resources from the conquered people, in particular gold and silver metals. Therefore, we proposed an approximation to understanding the spatial organization of the New Spain based on the use of pack animals that define the land movement. In addition, the technical contribution of this study is related to use an interdisciplinary formulation based on a computer code that joints different disciplines based on a set of sequential instructions.

This paper is organized as follows. The Material section describes the geospatial data related to the topography of the territory, the location of ancient harbors, and the up-to-date road network. In addition, we outline the computational libraries describing the open code for collecting, analyzing, and reports data and findings. The Method section shows the interdisciplinary approach describing the sequential process to compute and analyze the data. The Results section shows the findings based on maps, figures, and tables. Finally, the discussion section points out some items to be considered in the analysis and gives the conclusions and recommendations.

## Materials

**Fig. 1:**
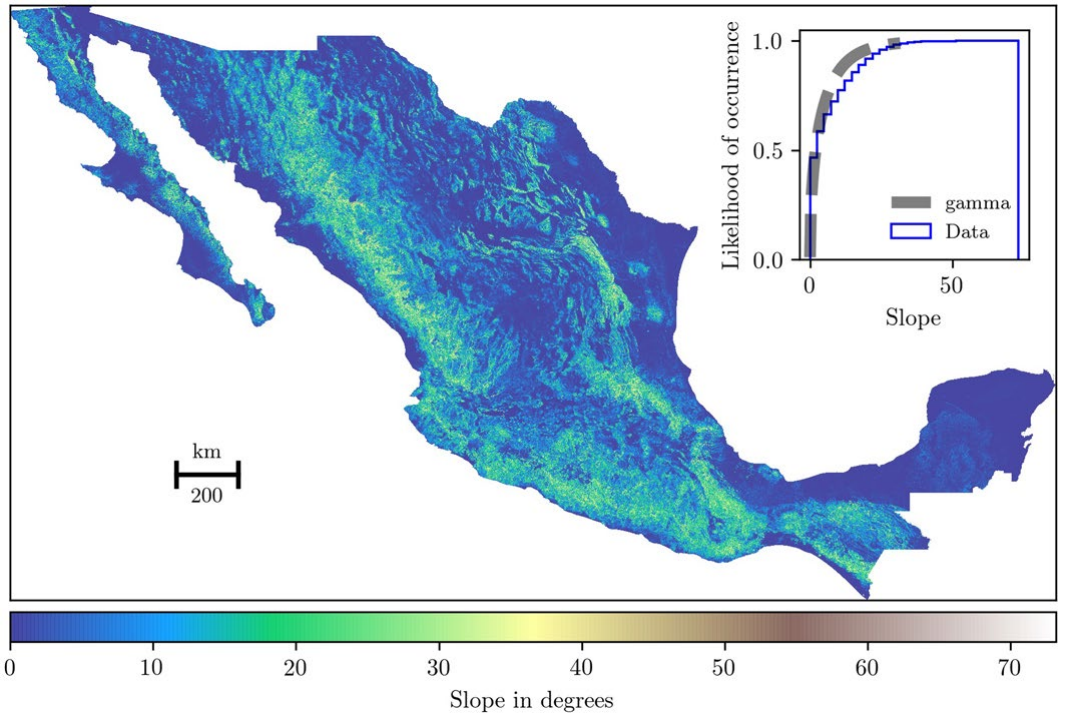
Slopes and its best-fit in the region. The gamma distribution best described the slope data. This distribution uses a probability density function of the form $f(x, \alpha ) = \frac{x^{\alpha -1} exp(-x)}{\Gamma (\alpha )}$, for $x \ge 0$, $\alpha > 0$ (see Table 1). The figure was generated using Python 2.7.15. and different Python libraries into the Anaconda Software Distribution, Computer software, Version 2-2.4.0. Anaconda, Nov. 2016. Web https://anaconda.com (see the Open Science Framework, project Horseback Network in the Colonial Era at Mexico, file rasterData_horseback.ipynb).

We used materials associated with geospatial data, historical resources, and computational libraries. The geospatial data is based on raster and vector data that were obtained from the authorized Mexican database of the National Institute of Statistics and Geography (INEGI, in Spanish). We used the Digital Elevation Model (DEM) of the Mexican territory, resolution of 120 m, based on the INEGI database. This data was translated to slopes based on a resolution of 144 m due to the computational convenience in geoprocessing the LCP (Fig. 1).

**Table 1:** Statistics of the best fit in Fig. 1.

Best fit	n value	Estimated parameters	First moments
gamma	5,642,028	(0.4645, 0.0, 9.8635)	(1.9475, 4.5824, 45.1997, 2.9342, 12.9146)

Estimated parameters: (parameter1, loc, scale). First moments: (median, mean, variance, skewness, kurtosis). Estimated parameters were rounded to four digits (estimated values can be found in the Open Science Framework, project Horseback Network in the Colonial Era at Mexico, Method).

**Fig. 2:**
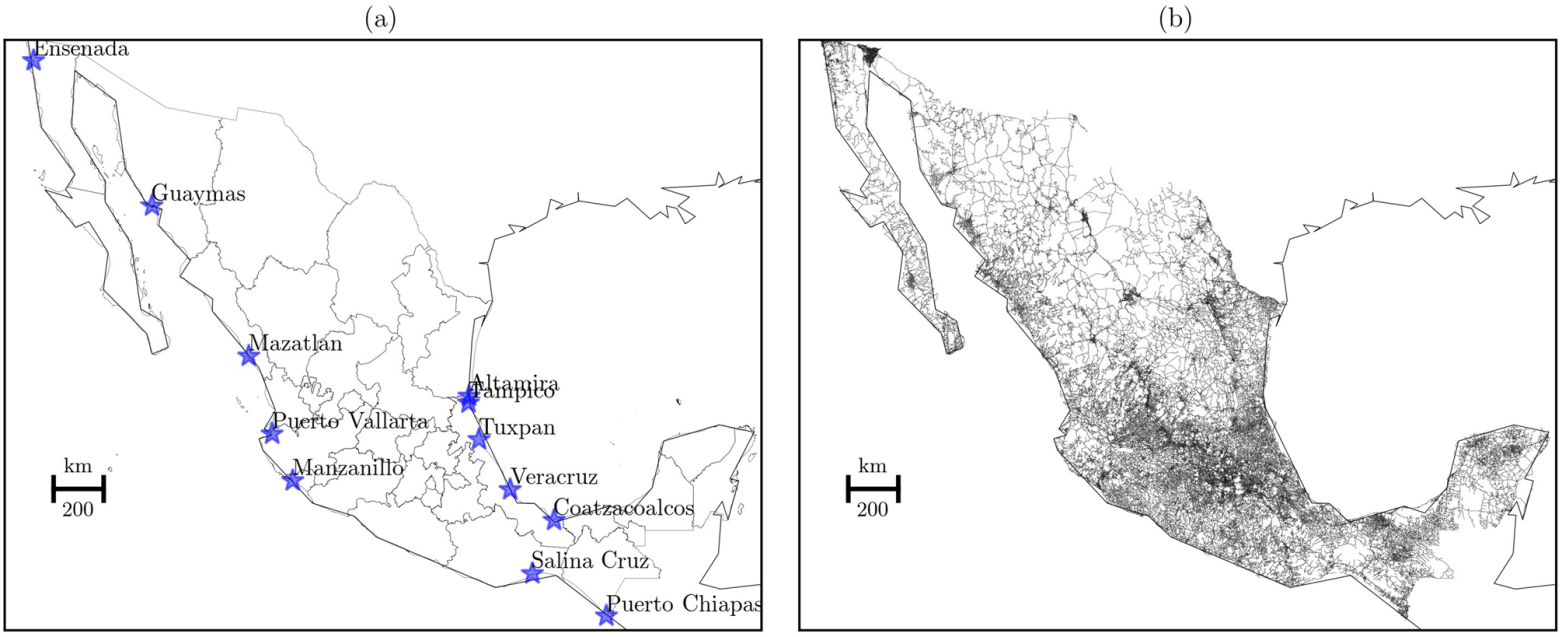
Vector data. **(a)** Ports associated with ancient harbors, and **(b)** road network. The figure shows the current names of ports and the up-to-date road network in the region. The figure was generated using Python 2.7.15. and different Python libraries into the Anaconda Software Distribution, Computer software, Version 2-2.4.0. Anaconda, Nov. 2016. Web https://anaconda.com (see the Open Science Framework, project Horseback Network in the Colonial Era at Mexico, file plotting_Material.ipynb).

Next, we used two types of vector data (Fig. 2). Subfigure (a) shows ports associated with ancient harbors based on the work of Lugo et al.^11^. It indicates important locations that functioned as natural harbors in the Postclassic period (900–1521 CE). Subfigure (b) shows the current road network. It shows the highest development of the land network suggesting the fastest way to connect places. We used it as the control dataset when comparing results of the NBC^19^.

Finally, we used different Python libraries to compute and analyze the above data. In particular, we used matplotlib^20^, basemap^21^, networkx^22^, numpy^23^, and gdal^24^. Based on these libraries, we generated code that we share in the Open Science Framework associated with our study because we embrace the transparency, openness, and reproducibility as features of science^25^. The section below describes the interdisciplinary method based on the complex systems approach that considers a sensitivity analysis and validation of models.

## Methods

The following method provides one approximation to identify where animal movement—leading by humans—may have taken place and connected ancient harbors in the region. The method is divided as two sections. The first section is associated with the path modeling in Archaeology—cost definition, accumulated cost surface, and LCP calculation. The second section is related to the complex network approach—generate a full network of paths, compute the node betweenness centrality, and compared the LCP formulations with the current road network in the region.

In particular, the LCP method is a procedure for finding an optimal path between two locations—source and target points—on a continuum surface. Locations are related to the harbors (Fig. 2a) and the surface is a grid cell with a particular data. In our case, we used the DEM (Fig. 1) and transformed it in three cases. The cases are based on the slope-dependent approximations (see “Path modeling” subsection) that connect harbors. To connect harbors, we used the A pathfinding algorithm and computed one source to all targets. We iterate this process until we passed all harbors as sources. After computing the LCP per slope-dependent approach, we find every set of cells that minimizes the connections between two locations. Then, we transformed each optimal path to a linestring (vector data) and generate the spatial network. These networks are formed by the set of nodes that represent harbors—endpoints of paths—and the rest of nodes represent the joint between line segments in paths. The number of nodes and edges per spatial network depend on the computation of the LCP and the resolution of the grid. For example, larger cells or a low resolution of the grid produces small number of nodes and edges per path; and smaller cells or a high resolution of the grid produces large number of nodes and edges per path. Finally, we analyze these spatial networks with the betweenness centrality measure (see the Complex network subsection). Having defined what is the general procedure of the LCP, we will now move on to describe the slope-dependent approximations and the node betweenness centrality.

### Path modeling

The slope data is one of the best approximations to understanding the movement on different types of topography. Slopes and their computational variations are essential data to use the LCP on a DEM. In this case, we used three slope-dependent approximations associated with riding horses. The first is the Tobler’s cost function in which its horseback variation is defined as the following exponential function:

1 $$\begin{aligned} W = [6.0 e^{-3.5|(tan(s)+0.05)|}] 1.25 \end{aligned}$$

where *W* is the walking velocity (Km/h) and *s* is the slope angle. According to Tobler^17^, (1) has to be multiplied by 1.25 to describe a horseback approximation. This function was estimated by data associated with the work of Imhof^26^. Even tough this formulation was not clear about the source data of the Imhof’s cost diagram, we used it because it is the most popular cost function.

The next approximation is based on the work of Self et al.^15^. They used data of racehorses in a track and analyzed the relationship between gradients and speed (m/s). They found that two linear regressions best describe the downhill (slope $<0$) and uphill (slope $>0$) behavior of galloping and trotting horses. These formulations can be described by the following piecewise function:

2 $$\begin{aligned} f(s) = \left\{ \begin{array}{ll} \left| \frac{0.687}{tan (s)}\right| &{} \quad s < 0 \\ &{} \\ \left| \frac{-0.453}{tan (s)}\right| &{} \quad s > 0 \end{array} \right. \end{aligned}$$

where *f*(*x*) is the horseback velocity (m/s) and *s* is the angle of the slope. This function suggested that the speed of horses decreases in the two intervals, but in the case of the decline the speed was slower than that the incline.

The last formulation is based on the work of Eaton et al.^16^ in which they used a treadmill to analyze the correlation between gradients and speed in incline conditions. Self et al.^15^ provided the following formulation:

3 $$\begin{aligned} f(s) = \left| \frac{-0.488}{tan (s)} \right| \end{aligned}$$

where *f*(*x*) is the horseback velocity and *s* is the angle of the slope. Functions (2) and (3) suggested a constant metabolic cost, i.e., the highest speeds were achieved in the lower gradients of the two cases^15^.

Based on these formulations, we computed their reciprocal function. In this case, we defined *f*(*x*) as the function and 1/*f*(*x*) or $f(x)^{-1}$ as its reciprocal. This reciprocal is important because the computation of the LCP is based on it—the LCP algorithm requires the minimum value of cells in the array.

Having defined these functions, we used them to transform our DEM to a particular accumulated cost surface. We computed the three LCPs based on the Tobler, Self et al., and Eaton et al. approximations. Then, we generated spatial models that have to be transformed to networks—nodes and edges with spatial attributes.

### Complex networks

The complex networks formulation consolidates our approximation because we translated the LCP results to spatial networks for data analysis. The data analysis consists in comparing the network of LCP models with an empirical model. In this case, we use the up-to-date road network as the control model in the territory. Then, according to Lugo and Alatriste-Contreras^14^, we identified the statistical distribution functions per spatial model to describe large-scale properties of the network. One of these properties is related to the node betweenness centrality measure because it can identify relevant data related to the urban growth^27, 28^. In particular, it identifies possible route sections of land corridors and nodes that work as control locations between harbors.

The betweenness centrality quantifies the importance of a node or edge by the amount of information flowing through it^29^. In our case, we computed the node betweenness centrality that indicates the potential of route sections to control the flow of traveling between harbors. Because we do not have data of flows, we assumed a uniformed number of travels between all pair of nodes. Based on this data, we can locate important points and understand the distribution of the flow of goods and people in the region.

Based on the networkx library^30^, and giving a graph with a set of vertices and edges, *G*(*V*, *E*), the node betweenness centrality is defined as following:

4 $$\begin{aligned} c_{B}(v) = \sum _{s,t \in V}^{} \frac{\sigma (s,t | v)}{\sigma (s,t)} \end{aligned}$$

where $c_{B}(v)$ is the node betweenness centrality—*c* represents the centrality, *B* the betweenness, and *v* a node—*V* is the set of nodes in the network, (*s*, *t*) is the number of shortest paths from node *s* to node *t*, and (*s*, *t*|*v*) is the number of those paths passing through a node. Then, high centrality scores indicate the most probable congested node in the network. Finally, this measure was translated to geospatial data and displayed it as maps.

This section has attempted to provide a clear description of the LCP method and the data analysis based on our three formulations. The section that follows shows the results of the data analysis based on the sensitivity analysis and the validation of our spatial models.

## Results

**Fig. 3:**
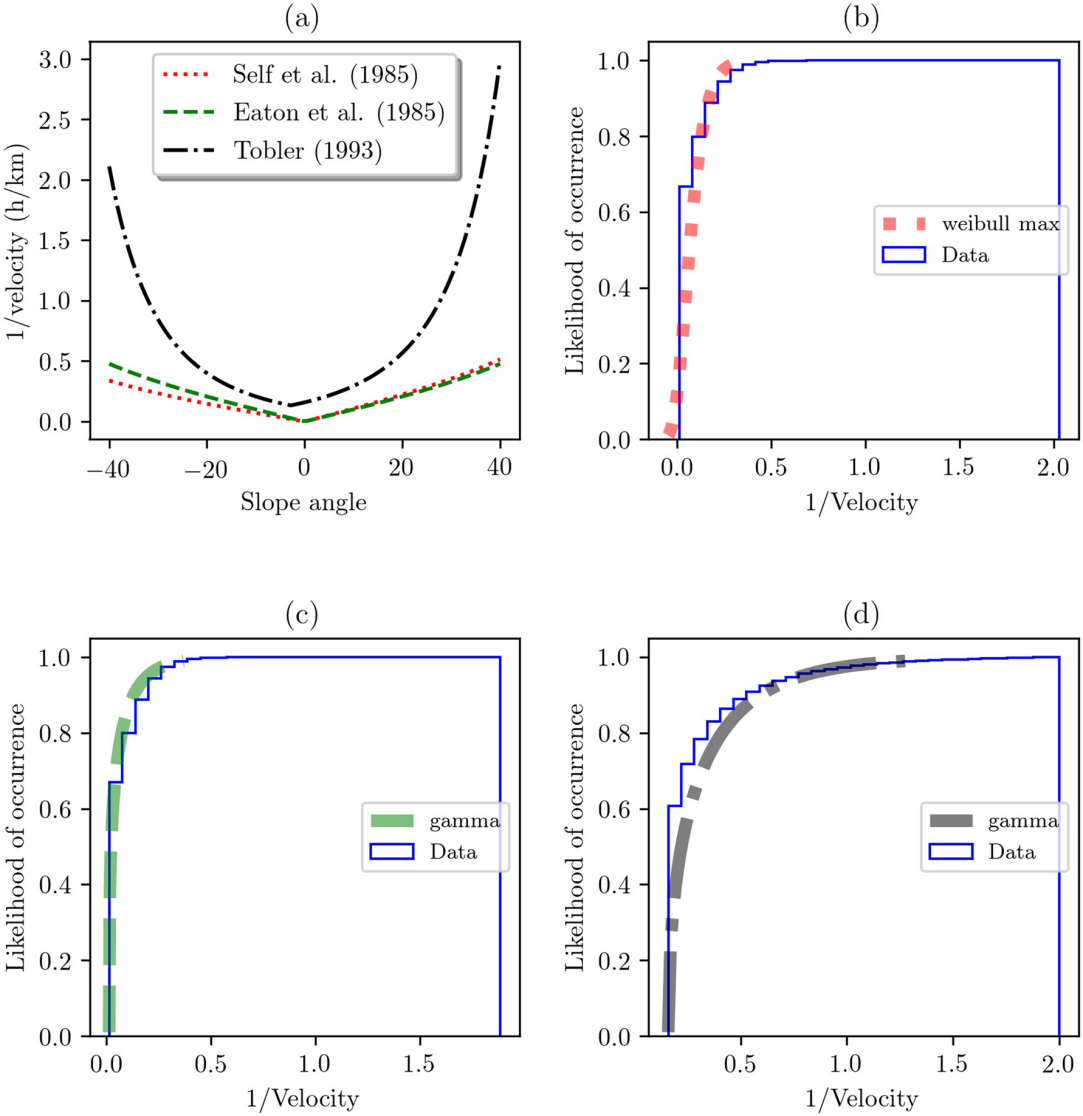
Three slope-dependent approximations of horseback riding. **(a)** Reciprocal of the slope-dependent approximations based on sample values, **(b)** best fit of the Self et al. approach, **(c)** best fit of the Eaton et al. formulation, and **(d)** best fit of the Tobler formula. Subfigure **(b)** is best described by a Weibull max distribution that shows a probability density function of the form $$ f(x, c) = c(-x)^{c-1} exp(-(-x)^{c})$$, for $$x < 0$$ and $$c > 0$$. Subfigures **(c)** and **(d)** are best describe by a gamma distribution that uses a probability density function of the form $$f(x, \alpha ) = \frac{x^{\alpha -1} exp(-x)}{\Gamma (\alpha )}$$, for $$x \ge 0$$, $$\alpha > 0$$ (see Table 2). Because of the rapid increase and decrease in values of velocity when the slope angle approaches to zero—vertical asymptote—in the three formulations, we controlled their maximum values by setting a limit of 76 km/h^15^. Above this limit, the velocity of horses is overestimated and therefore of no significance. In addition, in Tobler’s formulation, we controlled the maximum value of the reciprocal by setting a limit of 2 h/km because higher values of no significance can appear.

Our first result describes the behavior of horseback riding—cost definitions—in different slope formulations—cost surfaces (Fig. 3). This figure shows our three slope-dependent approximations based on their reciprocal that is associated with the array of slope angles. They indicate the effect of steepest routes on the velocity of horseback riding. As we can see, Self et al. and Eaton et al. approximations were similar in their reciprocal, subfigure (a), and their CDF values, but different in their best fit, subfigures (b) and (c). On the other hand, the reciprocal of Tobler’s shows a different behavior, subfigure (a), but similar best fit distribution than that of the Eaton et al. formulation, subfigure (d).

**Table 2:** Statistics of the best fit in Fig. 3.

Subfigure	n value	Best fit	Estimated parameters	First moments
(b)	5,642,028	Weibull max	(34.3567, 2.0529, 2.0093)	(0.0648, 0.0756, 0.0052, 0.9754, 1.6730)
(c)	5,642,028	Gamma	(0.2873, 0.0131, 0.1374)	(0.0221, 0.0526, 0.0054, 3.7309, 20.8799)
(d)	5,600,596	Gamma	(0.4350, 0.1588, 0.3581)	(0.2208, 0.3146, 0.0558, 3.0321, 13.7904)

Estimated parameters: (parameter1, loc, scale). First moments: (median, mean, variance, skewness, kurtosis).

Subfigure (a) indicates linear behaviors in the reciprocal of velocity related to the Self et al. and Eaton et al. formulations. In decline—positive values—there has been a steady rise when increasing the slope angle. On the other hand, in incline—negative values—there has been a steady decrease of slope angles showing slightly difference between them due to the computation of functions (2) and (3). On the other hand, the Tobler’s approach overestimates the reciprocal of velocity. Equation (1) reports a minimum value of reciprocal of 0.16 h/km and the maximum values vary exponentially. Therefore, these results seem to indicate that the Self et al. approach is the most significant and accurate method because its formulation was based on experimental data of racehorses.

**Fig. 4:**
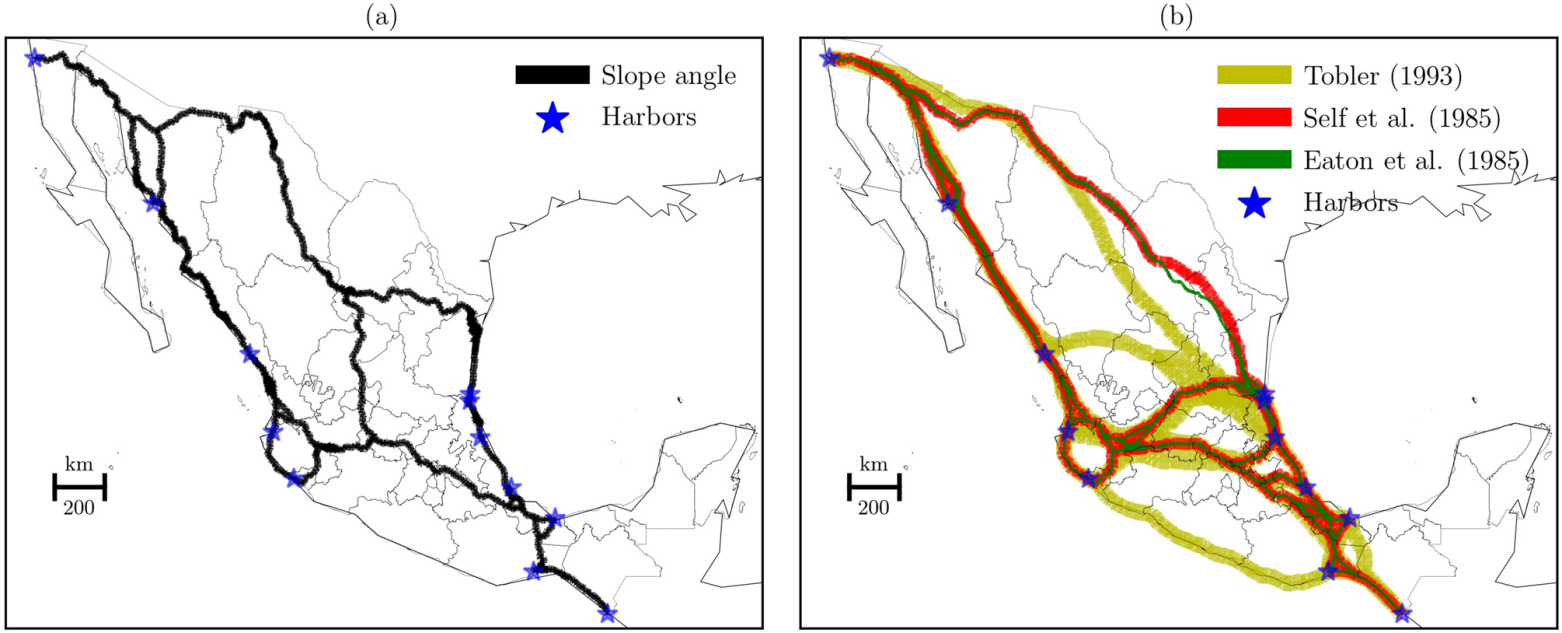
LCPs between ancient harbors. **(a)** Slope angle approximation, and **(b)** three slope-dependent formulations. These subfigures present the spatial networks of the transformed LCPs. The slope angle model shows 14,657 nodes and 22,508 edges; the Tobler approach shows 15,408 nodes and 23,257 edges; the Self et al. formulation shows 14,001 nodes and 21,255 edges; and The Eaton et al. approximation shows 14,114 nodes and 21,527 edges.

Let us now consider the reconstruction of paths between ancient harbors based on the above formulations (Fig. 4). For the purposes of comparison, this figure displays the LCP based on the slope angle, subfigure (a), and the LCPs based on the slope-dependent functions of riding horses, subfigure (b).

Comparing subfigure (a) and (b), we observed that the Self et al. and Eaton et al. formulations are more similar than the slope angle. This similarity is related to the linear transformation of the slope angle values in Eqs. (2) and (3). On the other hand, the Tobler formulation shows more options of paths that find routes across harsh terrains. This result is related to the transformation of the slope angle into the exponential equation (1). Therefore, the slope-dependent approaches show important variations in which the Tobler formulation decreases significantly the terrain roughness, meanwhile the Self et al. and Eaton et al. decrease moderately the surface roughness.

**Fig. 5:**
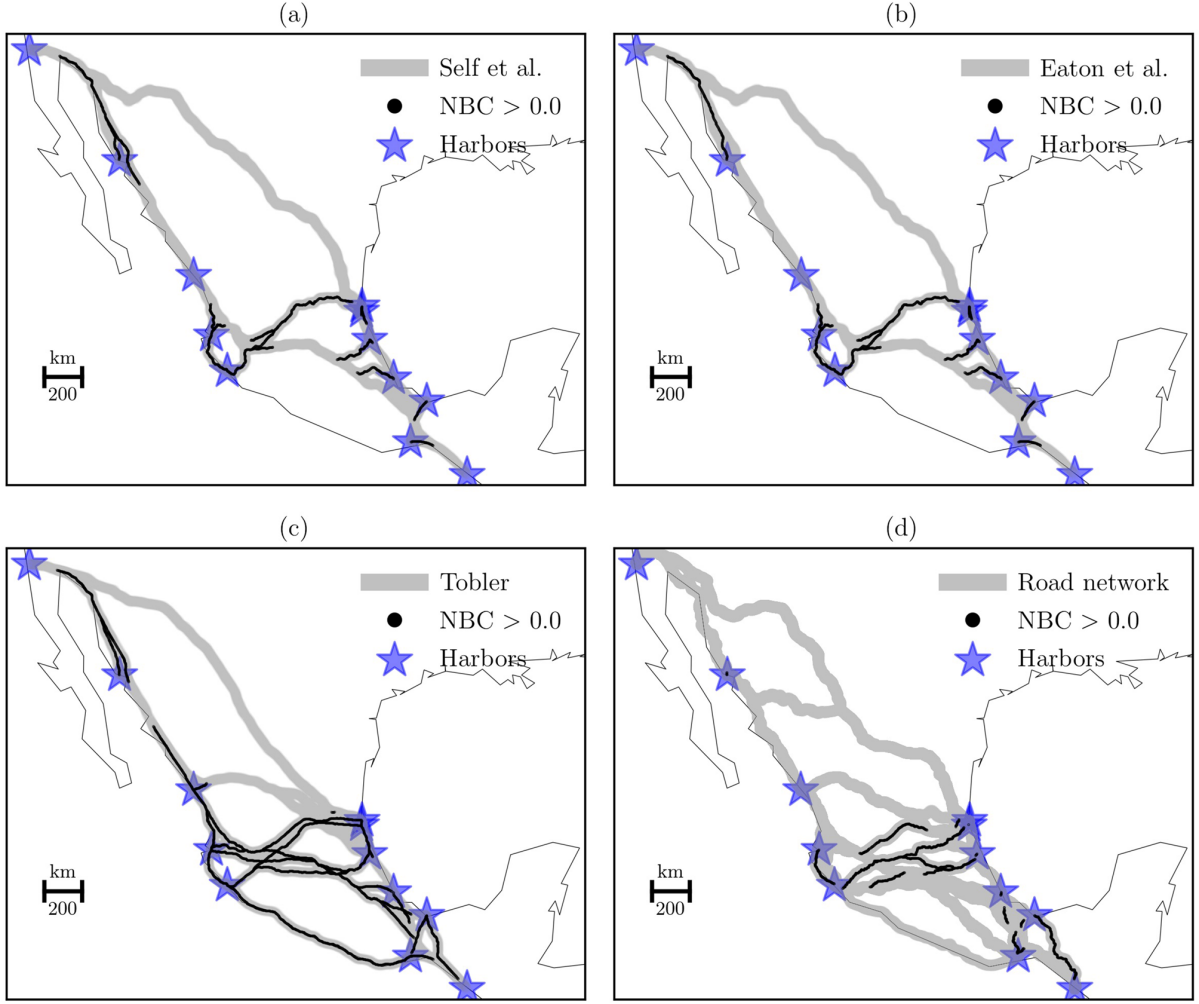
Spatial distribution of the NBC and the horseback formulations. **(a)** Self et al., **(b)** Eaton et al., **(c)** Tobler, and **(d)** road network. Subfigures **(a)**, **(b)**, and **(c)** are the training data, and subfigure **(d)** is the control data. The NBC used a histogram of 10 bins to show results. In this case, we display scores bigger than zero.

Next, we computed the NBC in our three formulations and compared them with the NBC of shortest paths based on the up-to-data road network between harbors (Fig. 5). Subfigures show the importance of nodes to control the flow of traveling per network formulation.

Figure 5 shows the potential of nodes by different horseback formulations to control the flow of traveling between harbors. Subfigures (a) and (b) show similar results suggesting that routes closer to Puerto Vallarta, Manzanillo, Tuxpan, Tampico, and Altamira harbors in the center of the region control the flow of traveling. Subfigure (c) shows a broad distribution of the NBC that covers the center and the south regions. This result suggests that most of the harbors are control points in the distribution of traveling flows. The subfigure (d) shows two patterns in the NBC that cross the territory. The first is related to the Puerto Vallarta, Manzanillo, Tuxpan, Tampico, and Altamira harbors located at the center of the region, and the second is associated with the southern harbors, Veracruz, Coatzacoalcos, Salina Cruz and Puerto Chiapas. Therefore, the NBC shows the importance of a set of nodes that could generate regions controlling the flow of traveling.

**Fig. 6:**
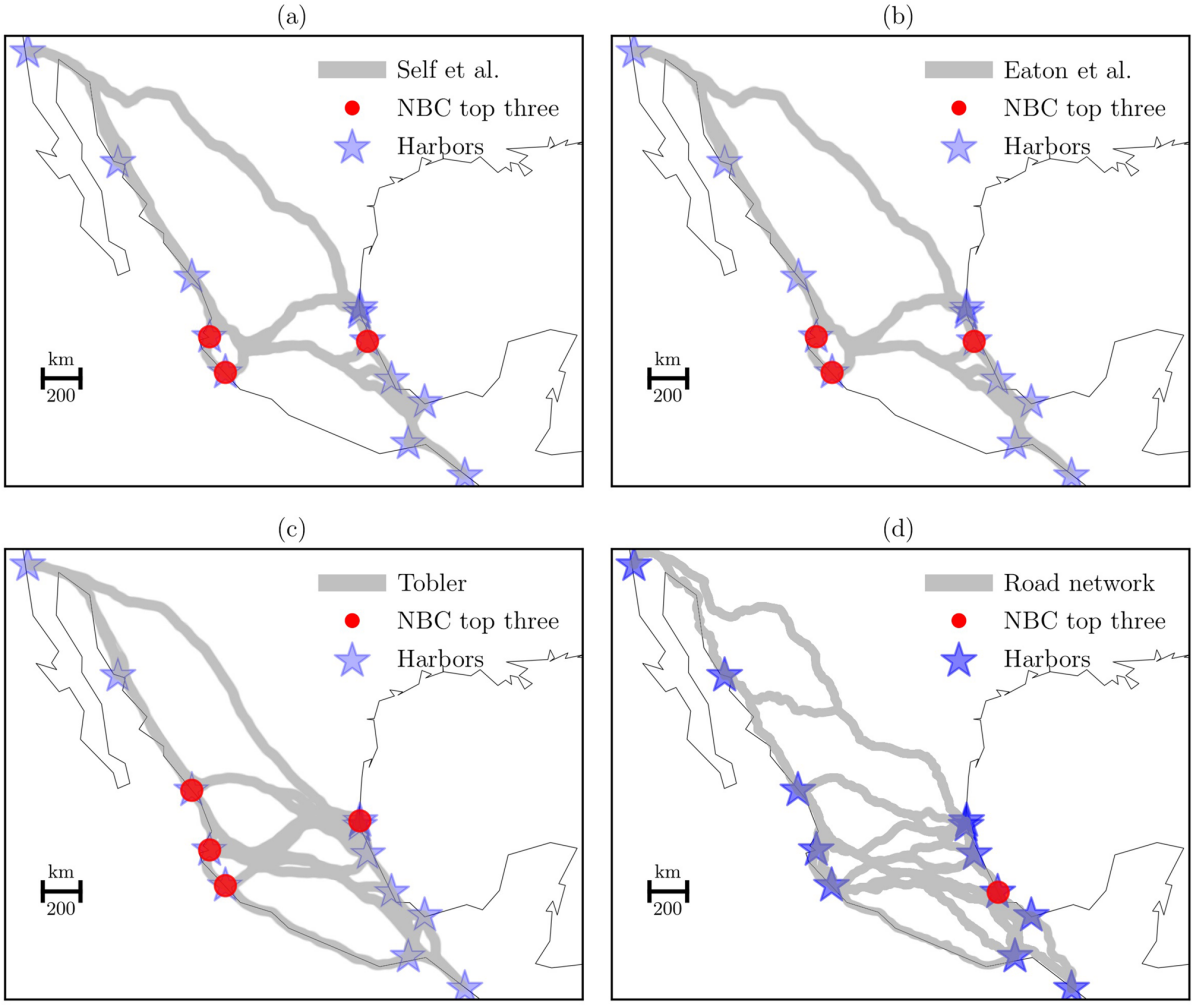
Spatial distribution of the top three NBC and the horseback formulations. **(a)** Self et al., **(b)** Eaton et al., **(c)** Tobler, and **(d)** road network. The top three NBC show the last three bins of a histogram of 10 bins using the NBC scores.

Another significant aspect of the NBC is to identify the most important nodes in the spatial networks (Fig. 6). Each subfigure shows the hierarchical nodes that are associated with harbors.

Figure 6 shows the spatial distribution of the highest scores of the NBC. Compared the subfigure (d) with the others, we can see the importance of fourth harbors in the horseback formulations. Subfigures (a) and (b) show the potential of Puerto Vallarta, Manzanillo and Tuxpan in the control of flow of traveling. In addition to these harbors, subfigure (c) introduces Mazatlan. It is evident that these results are in a good agreement with the LCP findings. However, subfigure (d) reveals the importance of Veracruz to control the current flow of traveling to all harbors. Therefore, we can assume the existence of the Veracruz harbor as one of the main locations to control the flow of horseback traveling in the beginning of the colonial period. These harbors could generate a region in the center of the territory in which the flow of goods and people were the key to control the New Spain.

**Fig. 7:**
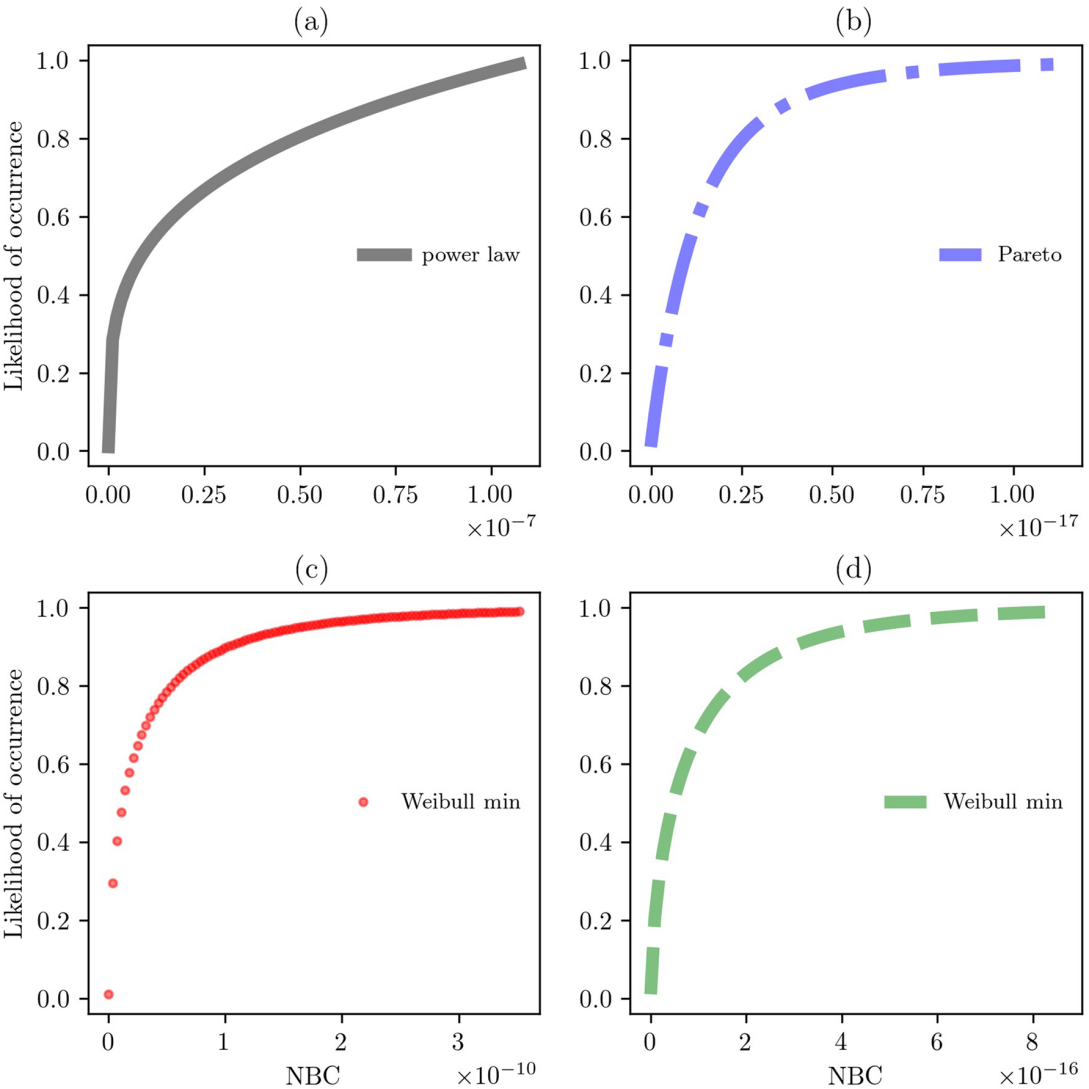
Estimated CDFs. **(a)** Self et al., **(b)** Eaton et al., **(c)** Tobler, and **(d)** road network. Subfigure **(a)** is best described by a power law distribution that shows a probability density function of the form $f(x, \alpha ) = \alpha x^{\alpha -1}$, for $0 \le x \le 1$, $\alpha > 0$. Subfigure **(b)** is best described by a Pareto distribution that shows a probability density function of the form $f(x, b) = \frac{b}{x^{b+1}}$, for $x \ge 0$ and $b > 0$. Subfigures **(c)** and **(d)** are best described by a Weibull min distribution that shows a probability density function of the form $f(x, c) = cx^{c-1} exp(-x^{c})$, for $x \ge 0$, $c > 0$ (see Table 3).

Finally, we show the large-scale attribute of each formulation based on its statistical distribution associated with the NBC (Fig. 7). Each CDF shows the estimated distribution that was identified based on the KS test for goodness of fit. CDFs show skewed behavior suggesting multiplicative mechanisms as a data generating process.

**Table 3:** Statistics of the best fit in Fig. 7.

Subfigure	n value	Best fit	Estimated parameters	First moments
(a)	7,973	Power law	(0.2711, 0.0, 0.0)	(0.0, 0.0, 0.0, 1.2896, 0.5058)
(b)	8,126	Pareto	(3.8513, 0.0, 0.0)	(0.0, 0.0, 0.0, 7.9018, NA)
(c)	9,966	Weibull min	(0.5614, 0.0, 0.0)	(0.0, 0.0, 0.0, 5.2140, 49.6778)
(d)	434,220	Weibull min	(0.6521, 0.0, 0.0)	(0.0, 0.0, 0.0, 3.9508, 26.8563)

Estimated parameters: (parameter1, loc, scale). First moments: (median, mean, variance, skewness, kurtosis). We rounded to four digits the columns of Estimated parameters and First moments (estimated values can be found in the Open Science Framework, project Horseback Network in the Colonial Era at Mexico, Method).

Figure 7 reveals that the common attribute of each network model is the skewed data based on the NBC. It signals a strong heterogeneity in networks in which there are few very central nodes associated with harbors (see Fig. 6). Comparing subfigures, there was different behaviors between subfigures (a) and (b), and there was a similar behavior between subfigure (c) and (d) because they show the same statistical distribution but different estimated parameters. This result demonstrates that the horseback paths of Tobler formulation share the same data generating process to the empirical road network when traveling between harbors. However, they differ in the location of the most central nodes. Therefore, these results suggest that these networks are organized in a hierarchical way in which harbors play the key role of distributing the flow of horseback traveling. The following part of this paper moves on to discuss those findings and conclude with final ideas.

## Discussion

The effect of horses in the traveling routes at the beginning of the colonial period was unprecedented. Not only horses, but also donkeys and mules controlled the flow of trade in the territory. In particular, harbors were connected to each other by horse riding based on a set of shortest paths that covered all the territory and crossed mainly the center of the region due to the location of the city of Tenochtitlan. These findings suggest that this region controlled and distributed the flow of traveling based on the harbors of Puerto Vallarta, Manzanillo, Tuxpan, and Veracruz. Therefore, the central region of Mexico was the key to control economically, politically, and militarily the New Spain.

Using the three slope-dependent approximations, the LCP findings showed different large-scale networks. We considered that the best of them is the Self et al. formulation because its horseback equation is based on a well-documented experimental data. On the other hand, the Eaton et al and Tobler approaches need more experimental results before been considered in future analyses. However, the shortest paths based on the Tobler’s formulation are similar to the shortest paths based on the up-to-date road network. It suggests that Tobler’s approach is consistent with the actual way of movement in the country. Therefore, the three approximations have to be used with caution due to the restricted and available experimental data between speed and slope of horses. Further investigations should generated new experimental data and find its best-fit estimations.

The NBC measure corroborates the presence of hierarchical nodes in the network models. Two training data, Self at al. and Eaton et al., show hierarchical nodes located in the center of the territory in which their most important nodes are associated with the harbors of Puerto Vallarta, Manzanillo, and Tuxpan. On the other hand, the Tobler’s training data presents an extended region of hierarchical nodes. However, their most important nodes are located in the central region, i.e., Mazatlan, Puerto Vallarta, Manzanillo, and Tampico. Finally, the control data provided evidence that the central region is tied to the harbor of Veracruz, which is the most important place in the up-to-date road network. Therefore, the central region of Mexico has been a vital area in controlling the movement of traveling.

Our complex systems approach revealed its advantages suggesting a way of comparing theoretical findings with empirical data. The former is the result of reproducing ancient conditions to generate some spatial patterns in spite of the existence of limited physical evidence. The latter represents the up-to-date of such patterns that could be related to the actual transportation system. This postulation improves the sensitivity analysis and the model validation of studies based on ancient transportation systems. Therefore, our study provides a framework for future studies to analyze complex problems based on an interdisciplinary approach. We provided a first approximation to understanding the connection between horseback routes and road networks in different time periods. They are located in the same region and terrain showing similar complex systems attributes. In particular, the NBC shows hierarchical nodes located in the central region that have controlled the flow of resources.

Finally, our study confirms the hypothesis in which the horse riding pathways connected all the Mexican territory based on ancient harbors, and the central region controlled the flow of traveling. However, in contrast to the walking pathways, the number of routes could be contracted, suggesting that the transport network avoided redundancies of route sections.

## Data Availability

The source code and the material and findings data of this study are openly available in the Open Science Framework, project Horseback Network in the Colonial Era at Mexico, at https://osf.io/y7vug/?view_only=95f396b591674b82b9de50d996d5e171 [doi].
